# Integrating atomistic molecular dynamics simulations, experiments, and network analysis to study protein dynamics: strength in unity

**DOI:** 10.3389/fmolb.2015.00028

**Published:** 2015-05-27

**Authors:** Elena Papaleo

**Affiliations:** Structural Biology and Nuclear Magnetic Resonance Laboratory, Department of Biology, University of CopenhagenCopenhagen, Denmark

**Keywords:** protein dynamics, molecular dynamics, protein NMR, enhanced sampling, metadynamics, protein structure

## Abstract

In the last years, we have been observing remarkable improvements in the field of protein dynamics. Indeed, we can now study protein dynamics in atomistic details over several timescales with a rich portfolio of experimental and computational techniques. On one side, this provides us with the possibility to validate simulation methods and physical models against a broad range of experimental observables. On the other side, it also allows a complementary and comprehensive view on protein structure and dynamics. What is needed now is a better understanding of the link between the dynamic properties that we observe and the functional properties of these important cellular machines. To make progresses in this direction, we need to improve the physical models used to describe proteins and solvent in molecular dynamics, as well as to strengthen the integration of experiments and simulations to overcome their own limitations. Moreover, now that we have the means to study protein dynamics in great details, we need new tools to understand the information embedded in the protein ensembles and in their dynamic signature. With this aim in mind, we should enrich the current tools for analysis of biomolecular simulations with attention to the effects that can be propagated over long distances and are often associated to important biological functions. In this context, approaches inspired by network analysis can make an important contribution to the analysis of molecular dynamics simulations.

## Protein dynamics and conformational changes and their importance in biology

It is now well-established that proteins, and biomolecules in general, are highly dynamic systems (Vendruscolo, 2007; Boehr et al., 2009; Zhuravlev and Papoian, 2010; Osawa et al., 2012). We moved, in the last decades, from the structure-function paradigm to the structure-dynamics-function triad, where not only the knowledge of the tertiary or quaternary assemblies is important to understand protein function, but also the dynamical behavior on different timescales (Henzler-Wildman and Kern, 2007; Klepeis et al., 2009; Villali and Kern, 2010; Wand, 2012). In this context, the knowledge of even protein states that account for a small population of the ensemble are important (Baldwin and Kay, 2009). Indeed, proteins undergo conformational changes even in their free and unbound/not modified state. Those minor conformations can resemble functional states, such as the structure that a protein assumes when it binds to another biological partner. The “perturbations” that affect the populations of the protein ensemble are caused by modifications, mutations, and interactions with other biomolecules and have impact on the related biological mechanisms (Popovych et al., 2006; Manley and Loria, 2012; Motlagh et al., 2012; Tsai and Nussinov, 2014).

## An atom-scale view on protein structure and dynamics by molecular dynamics simulations

Molecular simulations applied to biology are a powerful technique to integrate experimental studies, as attested by the Nobel Prize in Chemistry awarded to Martin Karplus, Micheal Levitt, and Arieh Warshel in 2013 for “the development of multiscale models for complex chemical systems.” Among the large array of simulation techniques that can cover the definition of a multiscale approach, I will here focus on atomistic classical molecular dynamics (MD) approaches. Other approaches that are important component of a multiscale approach are extensively covered in many review articles in the field (such as Rapaport, 1998; Koehl and Levitt, 1999; Warshel, 2003; Snow et al., 2004; Xia and Levitt, 2004; Karplus and Kuriyan, 2005; Sherwood et al., 2008; Schlick, 2010; Dror et al., 2012; Saunders and Voth, 2013; Salvatella, 2014; Field, 2015) and are not the focus of this perspective article.

Thirty-eight years are now passed since the first molecular dynamics (MD) simulation of a biomolecule, i.e., the bovine pancreatic trypsin inhibitor, was published (McCammon et al., 1977) and even if that was a short and in vacuum simulation, that work has a remarkable impact on the way in which we look at biomolecular structures. Thirty-eight years later, MD simulations are widespread tools that even experimentalists use to rationalize or guide experiments and a search in Pubmed with “molecular dynamics simulations” as a keyword provides more than 40,000 entries.

In MD methods, the system obeys to Newton's equation of motion and the interactions between atoms are described using, as physical models, molecular mechanics force fields (for a detailed discussion of the basis of MD see, for instance, Rapaport, 1998; Schlick, 2010; Kukol, 2015). Atomistic MD simulations have a unique strength of providing a description of biomolecules on several timescales from the femtosecond (fs) to the millisecond (ms), without renouncing to an atom-scale view for both the biomolecules and the solvent (Dror et al., 2012). They become over the last decades a useful method to integrate experimental research in structural biology and protein science, providing a “computational microscope” for proteins and their complexes (Dror et al., 2012).

## The importance of the force field parameters: improvements, limitations and experimental validation

The way in which we describe biomolecules in MD simulations depends on the force field, that we use to describe the system. Recently, new force fields have been developed that are in good agreement with many NMR-derived parameters, describing dynamics over different timescales (Best et al., 2012; Lindorff-Larsen et al., 2012; Huang and MacKerell, 2013; Reif et al., 2013). Despite the fact that the mathematical functional form of the classical force fields is similar, the new-generation force fields differ in parameters that are associated with a subset of very important torsional angles.

Providing the community with good and accurate force fields is an important task. Indeed, the risk to misinterpret the results from our simulations and, as a consequence, to provide biological models that are not meaningful is high. It is known that the even slight changes in force-field parameters have a large impact on the resulting conformational ensemble, as well as on the capability to reproduce experimental parameters (Lindorff-Larsen et al., 2012; Piana et al., 2014).

MD force fields, even if constantly improving, are still far from being perfect (Piana et al., 2014). Indeed, there are several aspects of protein dynamics that we are not able to simulate with accuracy, or even in a reasonable way. For example, currently employed classical MD force fields have been shown to overestimate salt bridges (Debiec et al., 2014). This observation can change how we interpret effects related to electrostatic interactions in proteins. Salt bridges, especially when they are in solvent-exposed positions, may be less populated in solution than expected from the analysis of MD simulations or X-ray structures, as convincingly argued by the NMR study of GB1 in solution (Tomlinson et al., 2009) or by the observation that mutations of residues involved in salt bridges in thermophilic or hyperthermophilic enzymes have often minor effects on thermal stability (Jónsdóttir et al., 2014). Moreover, classical force fields cannot accurately describe those interactions that need polarizable effects to be explicitly taken into account. This is crucial, for example, when we need to deal with metal-binding proteins (Banci, 2003). MD force fields also encounter the risk of over-compaction of the protein during the simulation, and this is especially important when we aim at studying systems as intrinsically disordered proteins (IDPs) or unfolded states (Knott and Best, 2012; Lambrughi et al., 2012; Invernizzi et al., 2013; Camilloni and Vendruscolo, 2014; Piana et al., 2014; Palazzesi et al., 2015). We encounter the risk, for example, to attribute a great importance to intramolecular interactions observed in MD-derived IDPs ensembles, which are a consequence of the highly compact states sampled during the simulation. Very recently, solutions have been provided for MD simulations of IDPs or unfolded states (Best et al., 2014; Piana et al., 2015). The authors of these works showed that it is possible to recover the compactness of IDPs or unfolded proteins observed, for example, by small-angle X-ray scattering thank to improved solvent models. This is an encouraging step toward a more accurate and reliable atomistic description of IDPs by simulations.

A continuous exchange between experimental biophysical techniques and MD is also needed.

Indeed, evaluation and validation of MD force fields cannot rely only on the study of few proteins, we need to move forward and test force fields on proteins with other folds. To do this, a large amount of experimental data are necessary for the comparison, along with methods to back-calculate from the simulated ensemble properties that can be measured experimentally, for instance by NMR spectroscopy or SAXS (Lindorff-Larsen et al., 2005; Lange et al., 2008; Kohlhoff et al., 2009; Sahakyan et al., 2011; Li and Brüschweiler, 2012; Camilloni and Vendruscolo, 2014). In this context, the usage of NMR parameters to cross-validate a MD ensemble should become a routine practice in computational biophysics.

## The coverage of the conformational space in MD simulations

On the other side, one of the main well-known issues of classical atomistic MD is the sampling of the conformational space. Indeed, during classical MD simulations, even if now we have the hardware (Friedrichs et al., 2009; Kohlhoff et al., 2014; Shaw et al., 2014) and software (Harvey et al., 2009; Pronk et al., 2013) to improve the performance and simulate even millisecond timescales, the protein encounters the risk to be trapped for a long time in a local basin of the conformational space, making the simulations not converging on even long timescales. Conventional MD simulations thus allow us to provide a limited description of protein dynamics since we neglect a major part of the conformational landscape. Also, we should keep in mind the risk to sample regions of the conformational space that are not relevant for the dynamics that we observe experimentally when a force field in bad agreement with the experimental data is used (Lindorff-Larsen et al., 2012).

The sampling problem has been recently overcome, for example, by enhanced-sampling techniques integrated to the atomistic force field description that MD provides. A rich portfolio of methods for enhancing sampling has been developed, each of them relying on different philosophies (Piana and Laio, 2007; Hritz and Oostenbrink, 2008; Sutto et al., 2012; Abrams and Bussi, 2013; Bernardi et al., 2014; Do et al., 2014; Papaleo et al., 2014; Spiwok et al., 2014; Barducci et al., 2015). Among them, metadynamics has the potential to enhance the sampling of rare transitions, which are often important in biology (Laio and Gervasio, 2008). Metadynamics approaches proved to be very accurate in recovering the conformational changes experimentally observed on the micro-millisecond time scale, which are the most important for many biological processes (Berteotti et al., 2009; Palazzesi et al., 2013; Sutto and Gervasio, 2013; Papaleo et al., 2014). In metadynamics, the sampling is enhanced along a properly selected set of reaction coordinates (i.e., collective variables) that have to account for the slowest degrees of freedom of the process of interest. It can be argued that it is limited to the usage of few collective variables at the time to ensure convergent simulations. The community, however, did extensive efforts in the last years to mitigate this problem, for instance, by reweighting procedures to calculate collective variables not used directly to bias the simulations (Bonomi et al., 2009) or by approaches as bias exchange metadynamics (Piana and Laio, 2007), in which the replicas exchange between different collective variables rather than in the temperature space.

The development of methods to predict NMR-derived parameters that are probes of dynamics over different timescales mentioned in the previous section, not only provides a solid strategy to evaluate the quality of MD ensembles but it also allow to simulate the protein integrating the atomistic description of the MD force fields with the experimental data. In this context, the usage of NMR parameters to restrain MD simulations is becoming a popular application and provided encouraging results toward a more complete picture of the complex dynamics of proteins (Lindorff-Larsen et al., 2005; Tang et al., 2007; Vendruscolo, 2007; Fenwick et al., 2011; Camilloni et al., 2012, 2013; Camilloni and Vendruscolo, 2014). In some of these approaches, the restraints are generally averaged over multiple replicas to better reflect the intrinsic average nature of the experimental data. These computational methods, among the enhanced sampling techniques that can be integrated to the atomistic MD force fields, have also the capability to recover the dynamics observed experimentally in the millisecond regime with high accuracy and without limiting the analysis to a small subset of collective variables, as metadynamics does.

## When the structural effects come from distal site: long-range communication in protein dynamics and network analysis applied to MD simulations

The network paradigm has been extensively used to describe structure, topology and dynamics of proteins (Vishveshwara et al., 2009; Atilgan et al., 2012; Collier and Ortiz, 2013; Csermely et al., 2013; Feher et al., 2014). The intramolecular non-covalent interactions in a protein are known to be crucial in determining the protein structure and they can be collectively represented in the form of a network, namely a Protein Structure Network (PSN), where the residues are the nodes of the network connected by edges that depend on their interaction strength. PSNs are “small worlds” (Vendruscolo et al., 2002; Atilgan et al., 2004) suitable for the fast transmission of conformational changes at distal sites. Indeed, in the small world of PSNs, the residues can communicate through the shortest paths available and multiple paths with common nodes are used (Del Sol et al., 2009). Several important issues, however, are, still unsolved when it comes to network analysis of protein ensembles. We can identify paths of communications between distal residues and we see that they are modified by perturbations. Very often, however, the way in which the native network of a protein is affected by a perturbation is not easy to predict. We do not exactly know if the paths that we calculate in a structural ensemble are really relevant for functional dynamics, or even for the conformational changes observed in the experiments. The community is collecting some examples in favor of a relationship between the networks observed in the protein structure and functional properties of proteins (Whitley and Lee, 2009; Mariani et al., 2013; Van den Bedem et al., 2013; Invernizzi et al., 2014; Papaleo et al., 2012b, 2014). We are, however, still far from being able to derive straightforward information and predictions out of the protein network. More efforts are certainly needed in this direction, also considering the potential that these approaches can have in applications such as drug discovery and protein engineering (Kar et al., 2010; Nussinov and Tsai, 2013).

Several methods are now available to identify networks and paths of communication in protein ensembles, such as the ones collected by MD simulations. The originally named PSN approach relies on the description of atomic contacts between residues that also feature correlated motions (Ghosh and Vishveshwara, 2007; Seeber et al., 2011; Papaleo et al., 2012a). It has become very popular also thanks to the availability of tools to derive PSN from both experimental and simulated ensembles (Seeber et al., 2011; Pasi et al., 2012; Bhattacharyya et al., 2013; Tiberti et al., 2014). The metrics to estimate correlated pairs of residues can range from Person correlation to Mutual Information and often suffer of the disadvantage in terms of convergence and statistical significance of the data. Caution has thus to be taken in deriving correlation maps from a MD ensemble and we should avoid in MD applications to biological target to use just a unique average correlation map from an entire MD trajectory. Other methods have been recently proposed and applied to MD simulations, relying on different philosophies, such as the usage of structural alphabets (Pandini et al., 2013), the integration of topology, correlated motions and communication propensity (Allain et al., 2014), the notion of energetic coupling between protein residues. (Ribeiro and Ortiz, 2014) or even the application of concepts inspired by engineering in which the protein is treated as a mechanical construct rather than a chemical molecule (Stacklies et al., 2011) with the possibility to trace communication even in stiff structures without evident atomic displacement.

The contribution of the solvent in the modulation of long-range communication in proteins should not be neglected and the community still lacks successful approaches to analyze the solvent-protein intermolecular networks, making this an appealing field for future research.

All the methods mentioned above often rely on the integration between network analysis and MD simulations. It is thus important to achieve a proper conformational sampling during the simulations, verify the reproducibility of the results over different replicates of the same system and o evaluate how long the simulations have to be extended to get meaningful results from network analysis. Indeed, long-range effects are often associated to conformational changes occurring on microsecond-millisecond timescales. Thus, the limitation inherent in the sampling achieved by classical MD has to be kept in mind. We recently showed that MD simulations of 100 ns or 1 μ s of the same system provided similar average PSN results (Invernizzi et al., 2014) but this account only for one case of study, making any generalization very hard.

## The strength is in unity: integration of molecular dynamics to experimental observable

In conclusion, we have reached now a point in which both from the computational and the experimental side, there are a large amount of information on protein dynamics and techniques to explore it on different timescale, as well as techniques to disclose effects that can be mediated by distal communicating sites and to actually describe these paths of communication at the atom-scale. The strength is now in the unity, in the capability to tightly integrate the computational and experimental approaches combining their own capabilities and overcoming their own limitations for a more detailed and accurate understanding of protein dynamics at the atomic level.

### Conflict of interest statement

The author declares that the research was conducted in the absence of any commercial or financial relationships that could be construed as a potential conflict of interest.

## References

[B1] Abrams C. Bussi G. (2013). Enhanced sampling in molecular dynamics using metadynamics, replica-exchange, and temperature-acceleration. Entropy 16, 163–199. 10.3390/e16010163

[B2] Allain A. Chauvot de Beauchêne I. Langenfeld F. Guarracino Y. Laine E. Tchertanov L. (2014). Allosteric pathway identification through network analysis: from molecular dynamics simulations to interactive 2D and 3D graphs. Faraday Discuss. 169, 303–321. 10.1039/C4FD00024B 25340971

[B3] Atilgan A. R. Akan P. Baysal C. (2004). Small-world communication of residues and significance for protein dynamics. Biophys. J. 86, 85–91. 10.1016/S0006-3495(04)74086-2 14695252 PMC1303839

[B4] Atilgan C. Okan O. B. Atilgan A. R. (2012). Network-based models as tools hinting at nonevident protein functionality. Annu. Rev. Biophys. 41, 205–225. 10.1146/annurev-biophys-050511-102305 22404685

[B5] Baldwin A. J. Kay L. E. (2009). NMR spectroscopy brings invisible protein states into focus. Nat. Chem. Biol. 5, 808–814. 10.1038/nchembio.238 19841630

[B6] Banci L. (2003). Molecular dynamics simulations of metalloproteins. Curr. Opin. Chem. Biol. 7, 143–149. 10.1016/S1367-5931(02)00014-5 12547439

[B7] Barducci A. Pfaendtner J. Bonomi M. (2015). Tackling sampling challenges in biomolecular simulations. Methods Mol. Biol. 1215, 151–171. 10.1007/978-1-4939-1465-4_8 25330963

[B8] Bernardi R. C. Melo M. C. R. Schulten K. (2014). Enhanced sampling techniques in molecular dynamics simulations of biological systems. Biochim. Biophys. Acta 1850, 872–877. 10.1016/j.bbagen.2014.10.019 25450171 PMC4339458

[B9] Berteotti A. Cavalli A. Branduardi D. Gervasio F. L. Recanatini M. Parrinello M. (2009). Protein conformational transitions: the closure mechanism of a kinase explored by atomistic simulations. J. Am. Chem. Soc. 131, 244–250. 10.1021/ja806846q 19067513

[B10] Best R. B. Zheng W. Mittal J. (2014). Balanced protein-water interactions improve properties of disordered proteins and non-specific protein association. J. Chem. Theory Comput. 10, 5113–5124. 10.1021/ct500569b 25400522 PMC4230380

[B11] Best R. B. Zhu X. Shim J. Lopes P. E. M. Mittal J. Feig M.. (2012). Optimization of the additive CHARMM all-atom protein force field targeting improved sampling of the backbone φ, ψ and side-chain χ (1) and χ (2) dihedral angles. J. Chem. Theory Comput. 8, 3257–3273. 10.1021/ct300400x 23341755 PMC3549273

[B12] Bhattacharyya M. Bhat C. R. Vishveshwara S. (2013). An automated approach to network features of protein structure ensembles. Protein Sci. 22, 1399–1416. 10.1002/pro.2333 23934896 PMC3795498

[B13] Boehr D. D. Nussinov R. Wright P. E. (2009). The role of dynamic conformational ensembles in biomolecular recognition. Nat. Chem. Biol. 5, 789–796. 10.1038/nchembio.232 19841628 PMC2916928

[B14] Bonomi M. Barducci A. Parrinello M. (2009). Reconstructing the equilibrium boltzmann distribution from well-tempered metadynamics. J. Comput. Chem. 30, 1615–1621. 10.1002/jcc.21305 19421997

[B15] Camilloni C. Cavalli A. Vendruscolo M. (2013). Replica-averaged metadynamics. J. Chem. Theory Comput. 9, 5610–5617. 10.1021/ct4006272 26592295

[B16] Camilloni C. Robustelli P. De Simone A. Cavalli A. Vendruscolo M. (2012). Characterization of the conformational equilibrium between the two major substates of RNase A using NMR chemical shifts. J. Am. Chem. Soc. 134, 3968–3971. 10.1021/ja210951z 22320129

[B17] Camilloni C. Vendruscolo M. (2014). Statistical mechanics of the denatured state of a protein using replica-averaged metadynamics. J. Am. Chem. Soc. 136, 8982–8991. 10.1021/ja5027584 24884637

[B18] Collier G. Ortiz V. (2013). Emerging computational approaches for the study of protein allostery. Arch. Biochem. Biophys. 538, 6–15. 10.1016/j.abb.2013.07.025 23933229

[B19] Csermely P. Korcsmáros T. Kiss H. J. M. London G. Nussinov R. (2013). Structure and dynamics of molecular networks: a novel paradigm of drug discovery: a comprehensive review. Pharmacol. Ther. 138, 333–408. 10.1016/j.pharmthera.2013.01.016 23384594 PMC3647006

[B20] Debiec K. T. Gronenborn A. M. Chong L. T. Fields F. (2014). Evaluating the strength of salt bridges – a comparison of current biomolecular force fields. J. Phys. Chem. B, 118, 6561–6569. 10.1021/jp500958r 24702709 PMC4064690

[B21] Del Sol A. Tsai C.-J. Ma B. Nussinov R. (2009). The origin of allosteric functional modulation: multiple pre-existing pathways. Structure 17, 1042–1150. 10.1016/j.str.2009.06.008 19679084 PMC2749652

[B22] Do T. N. Choy W.-Y. Karttunen M. (2014). Accelerating the conformational sampling of intrinsically disordered proteins. J. Chem. Theory Comput. 10, 5081–5094. 10.1021/ct5004803 26584388

[B23] Dror R. O. Dirks R. M. Grossman J. P. Xu H. Shaw D. E. (2012). Biomolecular simulation: a computational microscope for molecular biology. Annu. Rev. Biophys. 41, 429–452. 10.1146/annurev-biophys-042910-155245 22577825

[B24] Feher V. A. Durrant J. D. Van Wart A. T. Amaro R. E. (2014). Computational approaches to mapping allosteric pathways. Curr. Opin. Struct. Biol. 25, 98–103. 10.1016/j.sbi.2014.02.004 24667124 PMC4040315

[B25] Fenwick R. B. Esteban-Martín S. Richter B. Lee D. Walter K. F. A. Milovanovic D.. (2011). Weak long-range correlated motions in a surface patch of ubiquitin involved in molecular recognition. J. Am. Chem. Soc. 133, 10336–10339. 10.1021/ja200461n 21634390 PMC3686050

[B26] Field M. (2015). Technical advances in molecular simulation since the 1980s. Arch. Biochem. Biophys. [Epub ahead of print]. 10.1016/j.abb.2015.03.005 25772387

[B27] Friedrichs M. S. Eastman P. Vaidyanathan V. Houston M. Legrand S. Beberg A. L.. (2009). Accelerating molecular dynamic simulation on graphics processing units. J. Comput. Chem. 30, 864–872. 10.1002/jcc.21209 19191337 PMC2724265

[B28] Ghosh A. Vishveshwara S. (2007). A study of communication pathways in methionyl- tRNA synthetase by molecular dynamics simulations and structure network analysis. Proc. Natl. Acad. Sci. U.S.A. 104, 15711–15716. 10.1073/pnas.0704459104 17898174 PMC2000407

[B29] Harvey M. J. Giupponi G. Fabritiis G. De. (2009). ACEMD: accelerating biomolecular dynamics in the microsecond time scale. J. Chem. Theory Comput. 5, 1632–1639. 10.1021/ct9000685 26609855

[B30] Henzler-Wildman K. Kern D. (2007). Dynamic personalities of proteins. Nature 450, 964–972. 10.1038/nature06522 18075575

[B31] Hritz J. Oostenbrink C. (2008). Hamiltonian replica exchange molecular dynamics using soft-core interactions. J. Chem. Phys. 128, 144121. 10.1063/1.2888998 18412437

[B32] Huang J. MacKerell A. D. (2013). CHARMM36 all-atom additive protein force field: validation based on comparison to NMR data. J. Comput. Chem. 34, 2135–2145. 10.1002/jcc.23354 23832629 PMC3800559

[B33] Invernizzi G. Lambrughi M. Regonesi M. E. Tortora P. Papaleo E. (2013). The conformational ensemble of the disordered and aggregation-protective 182-291 region of ataxin-3. Biochim. Biophys. Acta 1830, 5236–5247. 10.1016/j.bbagen.2013.07.007 23891935

[B34] Invernizzi G. Tiberti M. Lambrughi M. Lindorff-Larsen K. Papaleo E. (2014). Communication routes in ARID domains between distal residues in helix 5 and the DNA-binding loops. PLoS Comput. Biol. 10:e1003744. 10.1371/journal.pcbi.1003744 25187961 PMC4154638

[B35] Jónsdóttir L. B. Ellertsson B. Ö. Invernizzi G. Magnúsdóttir M. Thorbjarnardóttir S. H. Papaleo E.. (2014). The role of salt bridges on the temperature adaptation of aqualysin I, a thermostable subtilisin-like proteinase. Biochim. Biophys. Acta 1844, 2174–2181. 10.1016/j.bbapap.2014.08.011 25172393

[B36] Kar G. Keskin O. Gursoy A. Nussinov R. (2010). Allostery and population shift in drug discovery. Curr. Opin. Pharmacol. 10, 715–722. 10.1016/j.coph.2010.09.002 20884293 PMC7316380

[B37] Karplus M. Kuriyan J. (2005). Molecular dynamics and protein function. Proc. Natl. Acad. Sci. U.S.A. 102, 6679–6685. 10.1073/pnas.0408930102 15870208 PMC1100762

[B38] Klepeis J. L. Lindorff-Larsen K. Dror R. O. Shaw D. E. (2009). Long-timescale molecular dynamics simulations of protein structure and function. Curr. Opin. Struct. Biol. 19, 120–127. 10.1016/j.sbi.2009.03.004 19361980

[B39] Knott M. Best R. (2012). A preformed binding interface in the unbound ensemble of an intrinsically disordered protein: evidence from molecular simulations. PLoS Comput. Biol. 8:e1002605 10.1371/journal.pcbi.1002605 22829760 PMC3400577

[B40] Koehl P. Levitt M. (1999). Theory and simulation. Can theory challenge experiments. Curr. Opin. Struct. Biol. 9, 155–156. 10.1016/S0959-440X(99)80021-9 10465610

[B41] Kohlhoff K. J. Robustelli P. Cavalli A. Salvatella X. Vendruscolo M. (2009). Fast and accurate predictions of protein NMR chemical shifts from interatomic distances. J. Am. Chem. Soc. 131, 13894–13895. 10.1021/ja903772t 19739624

[B42] Kohlhoff K. J. Shukla D. Lawrenz M. Bowman G. R. Konerding D. E. Belov D.. (2014). Cloud-based simulations on Google Exacycle reveal ligand modulation of GPCR activation pathways. Nat. Chem. 6, 15–21. 10.1038/nchem.1821 24345941 PMC3923464

[B43] Kukol A. (2015). Molecular Modeling of Proteins. New York, NY: Springer.

[B44] Laio A. Gervasio F. L. (2008). Metadynamics: a method to simulate rare events and reconstruct the free energy in biophysics, chemistry and material science. Rep. Prog. Phys. 71:126601 10.1088/0034-4885/71/12/126601

[B45] Lambrughi M. Papaleo E. Testa L. Brocca S. De Gioia L. Grandori R. (2012). Intramolecular interactions stabilizing compact conformations of the intrinsically disordered kinase-inhibitor domain of Sic1: a molecular dynamics investigation. Front. Physiol. 3:435 10.3389/fphys.2012.00435 PMC3504315 23189058

[B46] Lange O. F. Lakomek N.-A. Farès C. Schröder G. F. Walter K. F. A. Becker S.. (2008). Recognition dynamics up to microseconds revealed from an RDC-derived ubiquitin ensemble in solution. Science 320, 1471–1475. 10.1126/science.1157092 18556554

[B47] Li D.-W. Brüschweiler R. (2012). PPM: a side-chain and backbone chemical shift predictor for the assessment of protein conformational ensembles. J. Biomol. NMR 54, 257–265. 10.1007/s10858-012-9668-8 22972619

[B48] Lindorff-Larsen K. Best R. B. Depristo M. A. Dobson C. M. Vendruscolo M. (2005). Simultaneous determination of protein structure and dynamics. Nature 433, 128–132. 10.1038/nature03199 15650731

[B49] Lindorff-Larsen K. Maragakis P. Piana S. Eastwood M. P. Dror R. O. Shaw D. E. (2012). Systematic validation of protein force fields against experimental data. PLoS ONE 7:e32131. 10.1371/journal.pone.0032131 22384157 PMC3285199

[B50] Manley G. Loria J. P. (2012). NMR insights into protein allostery. Arch. Biochem. Biophys. 519, 223–231. 10.1016/j.abb.2011.10.023 22198279 PMC4086649

[B51] Mariani S. Dell'Orco D. Felline A. Raimondi F. Fanelli F. (2013). Network and atomistic simulations unveil the structural determinants of mutations linked to retinal diseases. PLoS Comput. Biol. 9:e1003207. 10.1371/journal.pcbi.1003207 24009494 PMC3757061

[B52] McCammon J. Gelin B. Karplus M. (1977). Dynamics of folded proteins. Nature 267, 585–590. 10.1038/267585a0 301613

[B53] Motlagh H. N. Li J. Thompson E. B. Hilser V. J. (2012). Interplay between allostery and intrinsic disorder in an ensemble. Biochem. Soc. Trans. 40, 975–980. 10.1042/BST20120163 22988850 PMC4231872

[B54] Nussinov R. Tsai C.-J. (2013). Allostery in disease and in drug discovery. Cell 153, 293–305. 10.1016/j.cell.2013.03.034 23582321

[B55] Osawa M. Takeuchi K. Ueda T. Nishida N. Shimada I. (2012). Functional dynamics of proteins revealed by solution NMR. Curr. Opin. Struct. Biol. 22, 660–669. 10.1016/j.sbi.2012.08.007 23000032

[B56] Palazzesi F. Barducci A. Tollinger M. Parrinello M. (2013). The allosteric communication pathways in KIX domain of CBP. Proc. Natl. Acad. Sci. U.S.A. 110, 14237–14242. 10.1073/pnas.1313548110 23940332 PMC3761601

[B57] Palazzesi F. Prakash M. K. Bonomi M. Barducci A. (2015). Accuracy of current all-atom force-fields in modeling protein disordered states. J. Chem. Theory Comput. 11, 2–7. 10.1021/ct500718s 26574197

[B58] Pandini A. Fornili A. Fraternali F. Kleinjung J. (2013). GSATools: analysis of allosteric communication and functional local motions using a structural alphabet. Bioinformatics 29, 2053–2055. 10.1093/bioinformatics/btt326 23740748 PMC3722520

[B59] Papaleo E. Lindorff-Larsen K. De Gioia L. (2012a). Paths of long-range communication in the E2 enzymes of family 3: a molecular dynamics investigation. Phys. Chem. Chem. Phys. 14, 12515–12525. 10.1039/c2cp41224a 22706570

[B60] Papaleo E. Renzetti G. Tiberti M. (2012b). Mechanisms of intramolecular communication in a hyperthermophilic acylaminoacyl peptidase: a molecular dynamics investigation. PLoS ONE 7:e35686. 10.1371/journal.pone.0035686 22558199 PMC3338720

[B61] Papaleo E. Sutto L. Gervasio F. L. Lindorff-Larsen K. (2014). Conformational changes and free energies in a proline isomerase. J. Chem. Theory Comput. 10, 4169–4174. 10.1021/ct500536r 26588555

[B62] Pasi M. Tiberti M. Arrigoni A. Papaleo E. (2012). xPyder: a PyMOL plugin to analyze coupled residues and their networks in protein structures. J. Chem. Inf. Model. 52, 1865–1874. 10.1021/ci300213c 22721491

[B63] Piana S. Donchev A. Robustelli P. Shaw D. (2015). Water dispersion interactions strongly influence simulated structural properties of disordered protein states. J. Phys. Chem. B 119, 5113–5123. 10.1021/jp508971m 25764013

[B64] Piana S. Klepeis J. L. Shaw D. E. (2014). Assessing the accuracy of physical models used in protein-folding simulations: quantitative evidence from long molecular dynamics simulations. Curr. Opin. Struct. Biol. 24C, 98–105. 10.1016/j.sbi.2013.12.006 24463371

[B65] Piana S. Laio A. (2007). A bias-exchange approach to protein folding. J. Phys. Chem. B 111, 4553–4559. 10.1021/jp067873l 17419610

[B66] Popovych N. Sun S. Ebright R. H. Kalodimos C. G. (2006). Dynamically driven protein allostery. Nat. Struct. Mol. Biol. 13, 831–838. 10.1038/nsmb1132 16906160 PMC2757644

[B67] Pronk S. Páll S. Schulz R. Larsson P. Bjelkmar P. Apostolov R.. (2013). GROMACS 4.5: a high-throughput and highly parallel open source molecular simulation toolkit. Bioinformatics 29, 845–854. 10.1093/bioinformatics/btt055 23407358 PMC3605599

[B68] Rapaport D. C. (1998). The Art of Molecular Dynamics Simulation. Cambridge, UK: Cambridge University Press.

[B69] Reif M. M. Winger M. Oostenbrink C. (2013). Testing of the GROMOS force-field parameter set 54A8: structural properties of electrolyte solutions, lipid bilayers, and proteins. J. Chem. Theory Comput. 9, 1247–1264. 10.1021/ct300874c 23418406 PMC3572754

[B70] Ribeiro A. A. S. T. Ortiz V. (2014). Determination of signaling pathways in proteins through network theory: importance of the topology. J. Chem. Theory Comput. 10, 1762–1769. 10.1021/ct400977r 26580384

[B71] Sahakyan A. B. Vranken W. F. Cavalli A. Vendruscolo M. (2011). Structure-based prediction of methyl chemical shifts in proteins. J. Biomol. NMR 50, 331–346. 10.1007/s10858-011-9524-2 21748266

[B72] Salvatella X. (2014). Understanding protein dynamics using conformational ensembles. Adv. Exp. Med. Biol. 805, 67–85. 10.1007/978-3-319-02970-2_3 24446357

[B73] Saunders M. Voth G. (2013). Coarse-graining methods for computational biology. Annu. Rev. Biophys. 42, 73–93. 10.1146/annurev-biophys-083012-130348 23451897

[B74] Schlick T. (2010). Molecular Modeling and Simulation: An Interdisciplinary Guide. New York, NY: Springer.

[B75] Seeber M. Felline A. Raimondi F. Muff S. Friedman R. Rao F.. (2011). Wordom: a user-friendly program for the analysis of molecular structures, trajectories, and free energy surfaces. J. Comput. Chem. 32, 1183–1194. 10.1002/jcc.21688 21387345 PMC3151548

[B76] Shaw D. E. Grossman J. P. Bank J. A. Batson B. Butts J. A. Chao J. C. (2014). Anton 2: raising the bar for performance and programmability in a special-purpose molecular dynamics supercomputer, in Proceedings of the International Conference for High Performance Computing, Networking, Storage and Analysis (New Orleans, LA: IEEE Press), 41–53.

[B77] Sherwood P. Brooks B. Sansom M. (2008). Multiscale methods for macromolecular simulations. Curr. Opin. Struct. Biol. 18, 630–640. 10.1016/j.sbi.2008.07.003 18721882 PMC6407689

[B78] Snow C. Sorin E. Rhee Y. Pande V. (2004). How well can simulation predict protein folding kinetics and thermodynamics? Annu. Rev. Biophys. Biomol. Struct. 34, 43–69. 10.1146/annurev.biophys.34.040204.144447 15869383

[B79] Spiwok V. Suæur Z. Hošek P. (2014). Enhanced sampling techniques in biomolecular simulations. Biotechnol. Adv. [Epub ahead of print]. 10.1016/j.biotechadv.2014.11.011 25482668

[B80] Stacklies W. Seifert C. Graeter F. (2011). Implementation of force distribution analysis for molecular dynamics simulations. BMC Bioinformatics 12:101. 10.1186/1471-2105-12-101 21501475 PMC3094238

[B81] Sutto L. Gervasio F. L. (2013). Effects of oncogenic mutations on the conformational free-energy landscape of EGFR kinase. Proc. Natl. Acad. Sci. U.S.A. 110, 10616–10621. 10.1073/pnas.1221953110 23754386 PMC3696764

[B82] Sutto L. Marsili S. Gervasio F. L. (2012). New advances in metadynamics. Wiley Interdiscip. Rev. Comput. Mol. Sci. 2, 771–779. 10.1002/wcms.110324079907

[B83] Tang C. Schwieters C. D. Clore G. M. (2007). Open-to-closed transition in apo maltose-binding protein observed by paramagnetic NMR. Nature 449, 1078–1082. 10.1038/nature06232 17960247

[B84] Tiberti M. Invernizzi G. Lambrughi M. Inbar Y. Schreiber G. Papaleo E. (2014). PyInteraph: a framework for the analysis of interaction networks in structural ensembles of proteins. J. Chem. Inf. Model. 54, 1537–1551. 10.1021/ci400639r 24702124

[B85] Tomlinson J. H. Ullah S. Hansen P. E. Williamson M. P. (2009). Characterization of salt bridges to lysines in the protein G B1 domain. J. Am. Chem. Soc. 131, 4674–4684. 10.1021/ja808223p 19281232

[B86] Tsai C.-J. Nussinov R. (2014). A unified view of “how allostery works”. PLoS Comput. Biol. 10:e1003394. 10.1371/journal.pcbi.1003394 24516370 PMC3916236

[B87] Van den Bedem H. Bhabha G. Yang K. Wright P. E. Fraser J. S. (2013). Automated identification of functional dynamic contact networks from X-ray crystallography. Nat. Methods 10, 896–902. 10.1038/nmeth.2592 23913260 PMC3760795

[B88] Vendruscolo M. (2007). Determination of conformationally heterogeneous states of proteins. Curr. Opin. Struct. Biol. 17, 15–20. 10.1016/j.sbi.2007.01.002 17239581

[B89] Vendruscolo M. Dokholyan N. Paci E. Karplus M. (2002). Small-world view of the amino acids that play a key role in protein folding. Phys. Rev. E Stat. Nonlin. Soft Matter Phys. 65, 1–4. 10.1103/PhysRevE.65.061910 12188762

[B90] Villali J. Kern D. (2010). Choreographing an enzyme's dance. Curr. Opin. Chem. Biol. 14, 636–643. 10.1016/j.cbpa.2010.08.007 20822946 PMC2987738

[B91] Vishveshwara S. Ghosh A. Hansia P. (2009). Intra and inter-molecular communications through protein structure network. Curr. Protein Pept. Sci. 10, 146–160. 10.2174/138920309787847590 19355982

[B92] Wand A. J. (2012). The dark energy of proteins comes to light: conformational entropy and its role in protein function revealed by NMR relaxation. Curr. Opin. Struct. Biol. 23, 75–81. 10.1016/j.sbi.2012.11.005 23246280 PMC3572299

[B93] Warshel A. (2003). Computer simulations of enzyme catalysis: methods, progress, and insights. Annu. Rev. Biophys. Biomol. Struct. 32, 425–443. 10.1146/annurev.biophys.32.110601.141807 12574064

[B94] Whitley M. J. Lee A. L. (2009). Frameworks for understanding long-range intra-protein communication. Curr. Protein Pept. Sci. 10, 116–127. 10.2174/138920309787847563 19355979 PMC2749244

[B95] Xia Y. Levitt M. (2004). Simulating protein evolution in sequence and structure space. Curr. Opin. Struct. Biol. 14, 202–207. 10.1016/j.sbi.2004.03.001 15093835

[B96] Zhuravlev P. I. Papoian G. A. (2010). Protein functional landscapes, dynamics, allostery: a tortuous path towards a universal theoretical framework. Q. Rev. Biophys. 43, 295–332. 10.1017/S0033583510000119 20819242

